# The Significance of Serum Immunoglobulin Concentrations in Children with Autism Spectrum Disorders: In Search of Potential Blood Biomarkers

**DOI:** 10.3390/ijms26189242

**Published:** 2025-09-22

**Authors:** Joanna Wawer, Agnieszka Chojęta, Genowefa Anna Wawer, Marcin Gładki, Aneta Klotzka, Bartłomiej Kociński, Tomasz Urbanowicz, Janusz Kocki, Ewelina Grywalska

**Affiliations:** 1 Department of Experimental Immunology, Medical University of Lublin, 20-059 Lublin, Poland; ewelina.grywalska@umlub.edu.pl; 2 Diagnostic Laboratory, University Children’s Hospital, 20-093 Lublin, Poland; agnieszka.chojeta@umlub.edu.pl; 3 Department of Foreign Languages, Medical University of Lublin, 20-059 Lublin, Poland; genowefa.wawer@umlub.edu.pl; 4 Department of Pediatric Cardiac Surgery, Poznan University of Medical Sciences, 61-701 Poznan, Poland; marcingladki@gmail.com (M.G.); wuket@wp.pl (B.K.); 5 1st Cardiology Department, Poznan University of Medical Sciences, 61-701 Poznan, Poland; aklotzka@onet.pl; 6 Cardiac Surgery and Transplantology Department, Poznan University of Medical Sciences, 61-701 Poznan, Poland; turbanowicz@ump.edu.pl; 7 Department of Clinical Genetics, Medical University of Lublin, 20-059 Lublin, Poland; janusz.kocki@umlub.edu.pl

**Keywords:** serum immunoglobulins, autism spectrum disorders, deregulation of the immune system

## Abstract

Autism spectrum disorders (ASD) are a heterogeneous group of neurodevelopmental disorders characterized by a number of dysfunctions in communication, social interactions and repetitive rigid patterns of behavior, interests, and activities. Despite much research, the causes of ASD remain elusive. In addition to genetic and epigenetic etiology, scientists have indicated inflammation, deregulation of cytokines, anti-brain autoantibodies, gut microbiota, and deregulated immunity as mechanisms possibly involved in the development of ASD phenotype. The aim of the study was to analyze the levels of IgA, IgE, and IgM immunoglobulins in the blood serum in patients with ASD to find out whether certain blood parameters are deregulated in that group of patients. The results suggest altered production of the immune cells in ASD patients which may be considered in the assessment of immune functions. Also, PCT% and LYMPH elevated values in patients with ASD might be of clinical relevance, possibly of predictive value for clinical preliminary diagnosis and therapy.

## 1. Introduction

Autism spectrum disorder (ASD) is a group of neurodevelopmental disorders characterized by a triad of symptoms, i.e., restrictive behavioral patterns, dysfunctional social deficits, and impaired verbal communication. Despite much research, the causes of ASD remain elusive. In addition to genetic and epigenetic etiology, scientists have indicated inflammation, deregulation of cytokines, anti-brain autoantibodies, gut microbiota, and impaired immunity as the mechanisms possibly involved in the development of ASD phenotype [1,2,3,4,5].

Understanding how immune system dysfunction can lead to behavioral changes in ASD requires understanding the complex network of interactions between different cell types of both the innate and adaptive immune systems. Some immunological factors modulate CNS functioning. Subpopulations of T and NK cells may exhibit altered activity and impair responses to stimuli [6,7]. Certain cytokines can inhibit neurogenesis and promote neuronal death, while others can promote the growth and proliferation of neurons and oligodendrocytes. Complement proteins and microglia may be involved in the brain’s elimination of additional neurons and synapses, which may increase neuronal transmission (synaptic pruning) in the process of synaptic scaling, i.e., the mechanism by which neurons can detect changes in the firing rate of impulses using a set of molecular sensors which regulate receptor trafficking to scale the accumulation of glutamate receptors at synaptic sites (synaptic scaling). In contrast, reactive brain autoantibodies can alter neuronal development or function. When multiple components of the immune system are deregulated, neurodevelopmental and behavioral changes may occur [8,9,10,11]. The immune system is a series of complex mechanisms activated when the body has been invaded by pathogens. Antigens stimulate the immune system to produce antibodies whose task is to identify and neutralize antigens. When innate immunity is deficient, the more comprehensive adaptive immune response is activated. The adaptive immune system can recognize, eliminate, and remember pathogens [11,12,13,14,15,16].

Among the potential nongenetic causes of ASD, scientists point to the deregulation of the immune system observed both in autistic children and their family members. This is especially confirmed by family history showing signs of impairment, exposure to bacterial and viral infections, and abnormal cytokine and chemokine profiles. The normal immune system transfers maternal immunoglobulin G (IgG) antibodies to the newborn. High concentrations in the placenta begin around the 17th week of pregnancy, thus providing the immunologically naïve fetus with passive protection against pathogens. Maternal IgG antibodies are also transferred to the newborn during breastfeeding and persist in the newborn throughout early infancy [17]. IgG, IgM, and IgA against nine neuron-specific antigens were identified in the blood serum of autistic children [18,19]. The obtained antibodies were combined with various CNS molecules exhibiting sequential homology with milk protein. The occurrence of brain-specific autoantibodies in some autistic children indicates that autoimmunity may play a role in the etiology of ASD [20]. Moreover, researchers suggest that children with ASD can develop hypogammaglobulinemia (low total immunoglobulin G (IgG)) which suggests that lower levels of IgG might be associated with more severe aberrant behaviors in children with ASD [21].

The first evidence for autoimmune etiology of ASD was reported in 1971 in a child with ASD with a strong family history of autoimmune disorders [22]. The authors observed that ASD shares many of the features typically recognized in autoimmune disorders. The epidemiological links between autoimmunity and ASD are compelling, and the similarities have spurred several investigators to connect biologically rooted autoimmune disorders with behaviorally defined ASD [23]. What is more, the presence of antibodies directed against elements of the CNS in the serum of autistic children indicates an autoimmune process which may be related to the pathology of some ASD cases [18]. Significantly higher numbers of autoantibodies are found in patients with ASD compared to healthy controls, but the pathophysiological significance of these antibodies detected in children with ASD is not well understood. The findings of autoimmunity in ASD families with excess anti-brain antibodies suggest that, in some patients, anti-CNS autoantibodies may aggravate or pathologically affect neuronal development in autistic children [18]. Epidemiological studies have shown an association between maternal autoimmune disease (rheumatoid arthritis, psoriasis, and celiac disease) and the subsequent risk of having a child with ASD [23,24,25].

Moreover, a sharp increase in the incidence of autoimmune diseases among families with ASD has been observed [26]. Researchers have associated ASD with personal or family history of autoimmune diseases such as diabetes, celiac disease, autoimmune thyroiditis, rheumatoid arthritis, psoriasis, and systemic lupus erythematous [1,27]. Post-mortem analysis of brain tissues from people with ASD revealed two main disrupted biological pathways: down-regulation of genes related to synaptic function and up-regulation of genes related to the immune system [28,29,30]. Alterations in the peripheral immune system with quantitative and qualitative immune dysfunction, particularly abnormal lymphocyte subpopulations, have also been reported [27].

Studies on the gut–brain axis have demonstrated that the human ‘second brain’, i.e., the enteric nervous system (ENS), communicates with the central nervous system (CNS) via the vagus nerve through the autonomic nervous system, ENS, neurotransmitters, hormones, and immune responses. Neurotransmitters produced in the gut affect emotions by regulating the gut–brain axis. The human gut microbiome is responsible for the maintenance of gut homeostasis, and thus brain functioning [31]. Experiments showed that the maternal gut microbiota promotes healthy brain development of her baby by regulating biochemical profiles and selecting metabolites in the fetal brains. Studies confirm the association between the gut–brain axis and ASD [32]. Microbiota dysbiosis in pregnancy, type of delivery, antibiotics, and stress can eventually lead to gut microbiome dysbiosis and colonization of pathogenic microbes, which affects the CNS function by the production of neurotoxins [29].

The objective of the study was to analyze certain aspects of immunological profiles in children with ASD. We investigated blood parameters and the levels of IgA, IgE, and IgM immunoglobulins in the blood serum in patients with ASD as a potential source of blood biomarkers of ASD. Our focus was to analyze differences in IgA, IgE, IgM levels between ASD and control groups with reference to age/gender.

## 2. Results

Concomitant diseases in the group of patients with ASD found on clinical examination are presented in Table 1.

Table 2 presents blood count parameters in the group of patients with and without ASD.

The analysis of CBC found WBC, PCT, and Lymph were significantly increased in the patients with ASD compared to the subjects without ASD.

The levels of immunoglobulins assessed in the blood serum in the examined groups are presented in Table 3, Table 4 and Table 5 below.

The results found no significant differences in the level of IgA, IgE, and IgM depending on the patient’s gender. However, a higher although statistically not significant (*p* < 0.05) level of IgA and IgE was observed in females (Table 3).

The analysis found no significant relationship between the age of patients and the level of IgA and IgE. However, it was noted that the level of IgM was higher (*p* < 0.05) in 14-year-old patients compared to 2- and 11-year-old patients (Table 4).

The levels of IgE and IgM were higher in patients without ASD. A similar although statistically not significant relationship was observed for the level of IgA in patients with and without ASD (Table 5).

Considering the correlation between gender and ASD status, statistically more prominent differences were found between IgE levels in males with and without ASD. In the case of IgM, the analysis of variance showed that within both genders and ASD status, the level of the studied immunoglobulin was higher in the group without ASD (*p* < 0.05) (Table 5).

The analysis of linear correlation r-Pearson found positive correlation between IgA level and age of the studied patients (Table 6). The values of IgE and IgM also increased, however they were statistically not significant.

## 3. Discussion

Patients with ASD present with a wide range of clinical symptoms including persistent deficits in communication and social interaction, and limited and repetitive behavior patterns, interests, or activities. These symptoms appear in early childhood and cause significant functional deficits. The comorbidities observed in our patients with ASD include anxiety, depression, attention deficit hyperactivity disorder, and epilepsy.

From a clinical point of view, the occurrence of comorbidities observed in ASD is important. There is a significant increase in the incidence of atopic diseases (including allergies and asthma) among patients with ASD [33]. If Th2 lymphocytes organize the immune response in atopic diseases, Tregs play a key role in maintaining tolerance to several antigens, thus playing a key role in allergy [33,34,35]. For example, a relative or complete defect of Tregs in the airways influences the Th2 immune response leading to allergic inflammatory diseases. Consistent with these data, an unstable Tregs phenotype was found in asthma patients, which was associated with more severe disease. These data indicate a central role for Tregs in atopic diseases. The researchers postulate that the decrease in Tregs observed in ASD patients may be at least partially responsible for this observed comorbid condition [34,35,36].

In addition, an increase in gastrointestinal symptoms (diarrhea/constipation) and intestinal permeability has long been observed in patients with ASD related to changes in the composition of microflora [31,37]. Numerous studies suggest that patients diagnosed with autism spectrum disorders suffer from numerous gastrointestinal disorders, i.e., symptoms like constipation, abdominal pain, diarrhea, and vomiting. Moreover, gut microbial dysbiosis has been implicated in the pathogenesis of inflammatory bowel disease (IBS), coeliac disease (CD), allergy, asthma, metabolic syndrome, cardiovascular disease, and obesity [29]. In our research, pervasive developmental disorders were also observed among the comorbidities in patients diagnosed with ASD; toxoplasmosis, atopic dermatitis, congenital cytomegalovirus, symptoms such as chronic diarrhea, allergic rashes, lactose intolerance, and a number of deficits related to the lack of independence in self-care, hyperactivity, lack of verbal contact, or auto-aggression.

Currently, various materials collected from patients, e.g., blood, fibroblasts and stem cells, cerebrospinal fluid, urine, and feces, are used for the analysis, which allows gaining insight into the pathophysiology and developing diagnostic biomarkers for ASD [4,5]. However, there are some limitations as to the choice of biological material. Our study used peripheral venous blood. That type of biological material can be used to assess the level of immune cells in the serum. However, it is not easy to gather a group of young patients with ASD, although it would seem that peripheral venous blood is an easily accessible diagnostic tool. Blood sampling is an invasive procedure. In the case of pediatric patients, especially autistic subjects, collecting blood is very difficult because they often show anxiety and/or fear, sometimes even phobias when taking blood and in contact with the foreign surroundings of the treatment room and medical staff [34,38]. Sometimes only a small amount of the collected blood samples is available. We managed to obtain blood samples from 40 patients with ASD; accordingly, 40 samples from children in the same age without ASD were collected.

In our study, the average number of leukocytes and lymphocytes was higher compared to the group without ASD. Similar observations were made by the researchers who compared the extracellular signal-related kinase (ERK) activation rate (the number of cytosolic-positive/nuclear-negative pERK lymphocytes divided by the total number of lymphocytes counted) in a group of patients with autistic disorders (13.8 ± 9.2 years) and neurotypical control groups (14.6 ± 9.4 years old). The authors suggest that increased lymphocyte activation in autism can be interpreted as a non-specific symptom of excessive cellular activity, able to be used as a biomarker capable of monitoring disease progression and even predict the course of treatment [39].

While the importance of platelet activation for blood clotting after vascular injury is well known, the studies have also shown that platelets are involved in patho/physiological processes observed in several diseases, e.g., cancer, inflammations, infections, and neurological diseases. The researchers suggest that platelets may serve as a peripheral biomarker or cellular model of autism as they share biological and molecular features with neurons, which would suggest a possible link between platelet markers and ASD. Platelets and neurons have similar characteristics; calcium-dependent mechanism of activation, and secretion of extracellular vesicles containing neurotransmitters and activating molecules such as serotonin, dopamine, epinephrine, glutamate, gamma-aminobutyric acid (GABA), calcium and ADP, and ATP (secreted after activation of 5-HT transporters (serotonin transporter [SERT] and vesicular monoamine transporter 2 [VMAT2]), and cell surface receptors such as receptors for the mentioned neurotransmitters. Some neuron-specific markers are expressed in platelets, e.g., reelin signaling protein and amyloid precursor protein [38]. We observed statistically increased levels of PCT [%] values in the group of patients with ASD compared to patients without ASD. However, the examined cohort was too small to allow for definitive conclusions in that respect.

Immunoglobulins (e.g., IgM, IgG, IgA, and IgE) encoded by the B cell receptor (BCR) genes are essential mediators of adaptive humoral immunity. The production of fetal IgM begins in early stages of gestation and increases substantially in the postpartum period. Endogenous IgG and IgA production, which requires B cell class-switching, remains limited until 6 months of age [40].

There is no agreement among researchers as to the levels of blood serum immunoglobulins in patients with ASD [37]. The patients with ASD were found to have increased IgG and IgM concentrations compared to healthy controls [41]. Contradictory results, i.e., low levels of immunoglobulin IgG and IgM in children diagnosed with ASD, were also observed. The researchers correlated their assessments with increased behavioral severity of autistic symptoms assessed by the Aberrant Behavior Checklist (ABC). They examined over 250 children and found reduced plasma IgG and IgM levels in young children with autism. Decreased IgG correlated negatively with scores on the ABC [21,42]. Moreover, the presence of autoantibodies was determined in ASD children by other researchers. The studies found increased plasma levels of the weakly active IgG4 subclass among ASD patients [43,44,45,46]. Also, lower IgG1 levels in the serum of boys with ASD with a similar negative correlation to communication assessed by the ADI-R were reported [5]. Other studies observed the shift in IgG subclass to IgG4 and suggested that children with autism are likely to have a higher percentage of circulating IgG with an inherently lower affinity for the antibody receptors on leukocytes [5,44]. Such different findings might be due to different sample sizes and age of patients with ASD, variations in types of control samples, and different experimental approaches. We observed significantly higher levels of IgE and IgM in patients without ASD. A similar although statistically not significant relationship was observed for the level of IgA in patients with and without ASD.

Immunoglobulins G are of particular interest in childhood disorders because their levels are very low at birth and it may take up to 10 years for certain isotypes to reach adult levels. They are part of the humoral immune response, activated in complex interactions between dendritic cells, T cells, and Ig-producing B cells. IgG levels are therefore a useful method to assess not only the development of immunity but also the immune functions in adolescence. Reduced IgG and IgM levels in children with autism have been observed. Researchers analyzed the relationship between blood IgG and IgM levels and the behavior of autistic subjects. The study did not show any difference in serum immunoglobulin concentration between the study group and the control group and between genders. They observed that the level of IgA immunoglobulin was lower in patients with autism and in males than in the group of healthy children. Scientists speculate that reduced IgA levels may be due to an autoimmune disease, impaired immune system function, or genetic defects in these children [47].

Our results found no significant relationship between the age of patients and the level of IgA and IgE. However, we noted that the level of IgM was increased in 14-year-old patients compared to 2- and 11-year-olds. IgM are the first antibodies that appear in contact with antigens (e.g., fungal, viral, bacterial, food, pollen). In addition, IgM plays a key role in humoral response, especially in initial stages of disease. Higher IgM levels may suggest ongoing infection, neoplasm, or hepatic disorder. Since the testing coincided with the beginning wave of COVID-19 pandemic, it might also suggest early stage of COVID-19. A Brazilian study on IgM found children and adolescents with positive serology had comorbidities in 32.2%, e.g., frequent atopy, respiratory, and cutaneous disorders. The analysis of IgM found a significant difference in the prevalence rate between children and adolescents (*p* = 0.003); children <9 years presented 7.3% of IgM positivity against 13.9% of adolescents [48].

## 4. Material and Methods

The study material consisted of samples of peripheral venous blood collected from minor patients with autism spectrum disorders (Genetic Clinic, Independent Public University Hospital No.4 in Lublin, Poland) and peripheral venous blood from minors without autism spectrum disorders (Diagnostic Laboratory, University Children’s Hospital in Lublin, Poland). The samples of peripheral venous blood were collected into EDTA test tubes by qualified medical personnel with strictly observed sterility.

The research project was approved by the Bioethics Committee of the Medical University of Lublin, Poland (decision no. KE-0254/175/2019 of 18 September 2019 and KE-0254/133/2021 of 27 May 2021). Consent was also obtained from the Head of the Diagnostic Laboratory University Children’s Hospital in Lublin, Poland. Also, children’s parents/legal guardians signed informed consent to use biological material for scientific research. Some patients (15 out of 55) were excluded from the study as their parents/legal guardians did not consent to participate, despite the information that biological material will be used anonymously and only for the research project.

The study group consisted of 40 minor patients with ASD, aged 2–14 years (peripheral venous blood). Control group were 40 minor patients without ASD, aged 2–14 years (peripheral venous blood). The peripheral venous blood was tested for CBC and the total serum concentrations of IgE, IgA, and IgM were determined. In the group with ASD only few patients (n = 6) had the level of IgG4 determined, so those results were not included into analysis. Antibody levels were determined by commercially available kits ELISA (Roche, Warsaw, Poland).

Statistical analysis was performed using Statistica v.13 software (StatSoft, TIBCO Software, Palo Alto, CA, USA). Comparison of blood counts in patients with ASD and without ASD was analyzed by Mann–Whitney U test, with *p* < 0.05 assumed statistically significant. Statistical differences between examined parameters were assessed using the analysis of variance (ANOVA) with Tukey’s HSD post hoc range test, *p* < 0.05. The correlations between the groups were determined by r-Pearson, *p* < 0.05.

## 5. Conclusions

Research into the relationship between immune system dysfunction and ASD has been conducted for over 40 years. The research data seem to support the hypothesis that immune deregulation may contribute in whole or in part to the development of autistic phenotypes often accompanied by various concomitant diseases and disorders.

The levels of immunoglobulin (though inconclusive), PCT%, and LYMPH elevated values in patients with ASD might be of clinical and possibly predictive value for preliminary diagnosis and therapy. Research suggests impaired immune response in the course of ASD and altered production of the immune cells. Moreover, peripheral venous blood can be a useful though not easily accessible biological material in case of ASD. Further research in larger cohorts is needed to enhance our comprehension of deregulated levels of antibodies and compromised immunity in ASD patients.

## Figures and Tables

**Table 1:** Concomitant diseases in the group of patients with ASD.

**Extended diagnosis/comorbidities**	ADHD, atopic dermatitis, allergy, childhood autism, pervasive developmental disorders, asthma, milk protein/lactose intolerance, diarrhea, family history (family member (sibling) affected), self-aggression, on gluten-free diet, without dysmorphic features
**Other deficits**	protruding tongue, slurred speech, selective eating, family member (sibling) affected, low level of self-care (cannot dress), use toilet (diapers), cannot clean the nose, diarrhea, waking up at night

**Table 2:** Comparison of blood counts in patients with ASD and without ASD.

	Patients with ASD	Patients Without ASD	*p*
**WBC [** **×10^9^/L]**	284.00	151.00	**0.01** *
**RBC [** **×10^12^/L]**	239.00	196.00	0.54
**HGB [g/dL]**	232.00	203.00	0.75
**HCT [%]**	246.00	189.00	0.35
**MCV [fL]**	203.00	232.00	0.336
**MCH [pg]**	201.50	233.50	0.30
**MCHC [g/dL]**	202.00	233.00	0.31
**RDW** **RBC [%]**	266.50	168.50	0.07
**HDW** **HGB [g/dL]**	182.50	93.50	0.87
**PLT [** **×10^9^/L]**	258.00	177.00	0.14
**PCT [%]**	277.50	157.50	**0.02** *
**PDW [%]**	267.00	168.00	0.06
**MPV [fL]**	200.50	234.50	0.28
**NEU [** **×10^9^/L]**	257.00	178.00	0.16
**LYMPH** **[×10^9^/L]**	289.00	146.00	**0.00** *
**MON [** **×10^9^/L]**	225.50	209.50	0.98
**EOS [** **×10^9^/L]**	222.00	213.00	0.89
**BASO** **[×10^9^/L]**	237.50	197.50	0.58
**NEU [%]**	206.00	229.00	0.40
**LYMPH [%]**	238.00	197.00	0.57
**MON [%]**	213.00	222.00	0.60
**EOS [%]**	202.00	−1.00	0.31
**BASO [%]**	205.00	−0.87	0.38
**LUC [%]**	175.00	−0.32	0.74
**LUC [** **×10^9^/L]**	180.00	0.00	1.00
**Total cholesterol**	211.00	0.88	0.37
**HDL**	151.00	−0.56	0.57
**Triglycerides**	187.50	−0.41	0.67
**LDL (calculated)**	141.50	0.13	0.88
**Not-HDL**	151.00	−0.56	0.57

* significant values.

**Table 3:** Mean blood serum IgA, IgE, and IgM in the female (F)/male groups (M).

Gender	IgA [mg/dL]	IgE [IU/mL]	IgM [mg/dL]
F	90.70	33.11	79.29
M	97.80	41.26	75.88

**Table 4:** Mean blood serum IgA, IgE, and IgM in age groups.

Age [Years]	IgA [mg/dL]	IgM [mg/dL]	IgE [IU/mL]
2	69.40	52.20	36.51
3	83.66	97.75	20.86
4	83.00	83.38	34.72
5	91.75	77.58	40.73
6	97.66	98.33	35.91
7	110.00	90.50	38.34
8	118.57	72.71	38.13
9	114.16	67.66	63.87
10	112.50	78.00	40.77
11	110.20	24.20	61.02
12	98.00	51.85	45.41
13	84.80	94.40	22.83
14	124.83	118.50 *	29.65

* *p* < 0.05.

**Table 5:** Blood serum IgA, IgE, and IgM in the groups of males (M) and females (F) with and without ASD.

Sex		IgA [mg/dL]		IgM [mg/dL]		IgE [IU/mL]	
	ASD Status	With ASD	Without ASD	With ASD	Without ASD	With ASD	Without ASD
**F**		79.33	94.97	46.53	91.57 *	17.30	39.04
**M**		87.62	101.06	41.06	86.98 *	14.84	49.72

* *p* < 0.05.

**Table 6:** Correlation of blood serum IgA, IgE, and IgM and patient’s age.

Variable	Blood Serum IgA [mg/dL]	Blood Serum IgE [IU/mL]	Blood Serum IgM [mg/dL]
Age [years]	0.28 *	0.06	0.05

* Positive correlation.

## Data Availability

The original contributions presented in this study are included in the article. Further inquiries can be directed to the corresponding author(s).
